# Highly conserved gene order and numerous novel repetitive elements in genomic regions linked to wing pattern variation in *Heliconius *butterflies

**DOI:** 10.1186/1471-2164-9-345

**Published:** 2008-07-22

**Authors:** Riccardo Papa, Clayton M Morrison, James R Walters, Brian A Counterman, Rui Chen, Georg Halder, Laura Ferguson, Nicola Chamberlain, Richard ffrench-Constant, Durrell D Kapan, Chris D Jiggins, Robert D Reed, William O McMillan

**Affiliations:** 1 Department of Biology, University of Puerto Rico – Rio Piedras, San Juan, Puerto Rico, USA; 2 Department of Ecology and Evolutionary Biology, University of California Irvine, Irvine, USA; 3 Department of Evolutionary and Functional Biology, University of Parma, Parma, Italy; 4 Program in Developmental Biology, Baylor College of Medicine, Houston, USA; 5 Department of Ecology and Evolutionary Biology, Cornell University, Ithaca, USA; 6 Department of Genetics, North Carolina State University, Raleigh, USA; 7 Department of Molecular and Human Genetics, Baylor College of Medicine, Houston, USA; 8 Human Genome Sequencing Center, Baylor College of Medicine, Houston, USA; 9 Department of Biochemistry and Molecular Biology, University of Texas – M.D. Anderson Cancer Center, Houston, USA; 10 Department of Zoology, University of Cambridge, Cambridge, UK; 11 FAS Center for Systems Biology, Harvard University, Cambridge, USA; 12 Centre for Ecology and Conservation, University of Exeter, Penryn, UK; 13 Center for Conservation Research and Training, University of Hawai'i at Manoa, Honolulu, USA

## Abstract

**Background:**

With over 20 parapatric races differing in their warningly colored wing patterns, the butterfly *Heliconius erato *provides a fascinating example of an adaptive radiation. Together with matching races of its co-mimic *Heliconius melpomene*, *H. erato *also represents a textbook case of Müllerian mimicry, a phenomenon where common warning signals are shared amongst noxious organisms. It is of great interest to identify the specific genes that control the mimetic wing patterns of *H. erato *and *H. melpomene*. To this end we have undertaken comparative mapping and targeted genomic sequencing in both species. This paper reports on a comparative analysis of genomic sequences linked to color pattern mimicry genes in *Heliconius*.

**Results:**

Scoring AFLP polymorphisms in *H. erato *broods allowed us to survey loci at approximately 362 kb intervals across the genome. With this strategy we were able to identify markers tightly linked to two color pattern genes: *D *and *Cr*, which were then used to screen *H. erato *BAC libraries in order to identify clones for sequencing. Gene density across 600 kb of BAC sequences appeared relatively low, although the number of predicted open reading frames was typical for an insect. We focused analyses on the *D- *and *Cr*-linked *H. erato *BAC sequences and on the *Yb*-linked *H. melpomene *BAC sequence. A comparative analysis between homologous regions of *H. erato *(*Cr*-linked BAC) and *H. melpomene *(*Yb*-linked BAC) revealed high levels of sequence conservation and microsynteny between the two species. We found that repeated elements constitute 26% and 20% of BAC sequences from *H. erato *and *H. melpomene *respectively. The majority of these repetitive sequences appear to be novel, as they showed no significant similarity to any other available insect sequences. We also observed signs of fine scale conservation of gene order between *Heliconius *and the moth *Bombyx mori*, suggesting that lepidopteran genome architecture may be conserved over very long evolutionary time scales.

**Conclusion:**

Here we have demonstrated the tractability of progressing from a genetic linkage map to genomic sequence data in *Heliconius *butterflies. We have also shown that fine-scale gene order is highly conserved between distantly related *Heliconius *species, and also between *Heliconius *and *B. mori*. Together, these findings suggest that genome structure in macrolepidoptera might be very conserved, and show that mapping and positional cloning efforts in different lepidopteran species can be reciprocally informative.

## Background

Among emerging evolutionary and ecological model organisms, the passion-vine butterfly genus *Heliconius *(Nymphalidae: Heliconiinae) offers particularly exciting possibilities for integrative research into the genetic and developmental basis of adaptive variation [1,2]. The genus, composed of around 40 species with hundreds of geographic variants, couples color pattern divergence with multiple cases of mimicry-related convergent evolution [2]. The wing color patterns of *Heliconius *are adaptations that warn potential predators of the butterflies' unpalatability [3] and also play an important role in speciation [4]. Nearly all *Heliconius *species participate in local Müllerian mimicry associations and, in any one area, the wing color patterns of different aposematic butterfly species converge into a handful (usually six or less) of clearly differentiated mimetic assemblages [5]. The color patterns characterizing many of these mimicry rings often change dramatically every few hundred kilometers. This pattern of convergent and divergent evolution in *Heliconius *is best exemplified by the mimetic relationship between *H. erato *and *H. melpomene*. The two species are distantly related within the genus and never hybridize [2,6,7], yet, where they co-occur, local races possess nearly identical wing patterns and have undergone parallel and congruent radiations into over 20 geographic races [5,8].

The multiple radiations of mimetic color patterns, particularly the parallel radiations of *H. erato *and *H. melpomene*, provide "natural experiments" for comparative studies into the genetic and developmental basis of adaptive change. In this paper, we describe a simple strategy that integrates growing genomic resources in *Heliconius *to identify regions of the genome near the loci that modulate wing pattern variation in *H. erato*. Our strategy relies on the fact that large phenotypic differences within species are caused by a handful of major effect loci [8] and that crosses can be designed that allow researchers to unambiguously follow the segregation of alleles at these loci [9,10]. By scanning through thousands of AFLP polymorphisms in these crosses we can identify markers tightly associated with particular color pattern genes. These markers are then used to probe newly available Bacterial Artificial Chromosome (BAC) libraries and allow us to obtain large sections of genomic sequence around color pattern genes. These targeted genomic sequences provide the first insights into the architecture of the *H. erato *genome including details on gene density, repeat structure and, with sequence information from homologous regions of the *H. melpomene *genome, the preservation of fine-scale gene order between the two co-mimics. These data facilitate comparative mapping work on the genetic basis of color pattern variation and convergence in *Heliconius*, including efforts to positionally clone the color pattern genes themselves. These data also provide some of the first information on patterns of microsynteny in lepidopteran genomes, complementing recent work showing marked patterns of synteny conservation at a macro scale between *H. melpomene *and the silk moth *Bombyx mori *[11].

**Figure 1:**
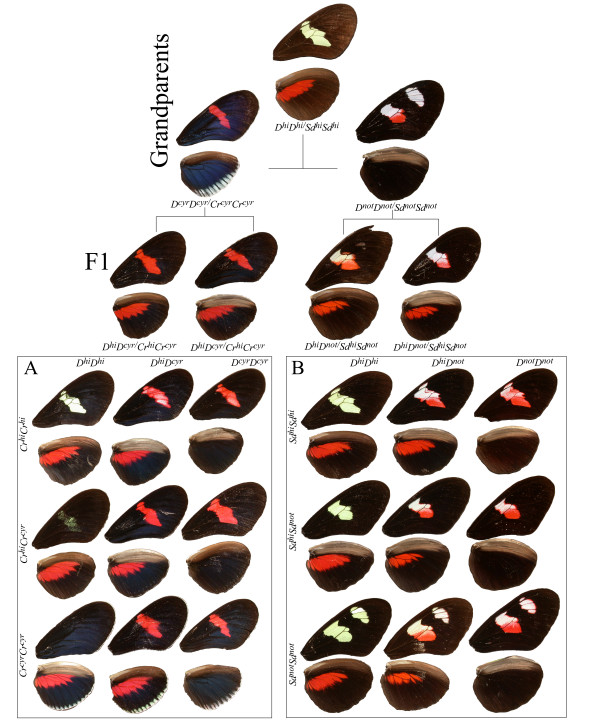
**Cross design and wing phenotypes**. Color pattern phenotypes observed in crosses between 'grand-parental' *H. himera *(middle) and *H. erato cyrbia *(left) and *H. erato notabilis *(right) resulting in two pairs of F1 parents with females on left, males on right. Each pair of F1 parents produced F2s with *H. himera *× *H. erato cyrbia *offspring (A) and the *H. himera *× *H. erato notabilis *offspring (B). In both F2 families, the observed phenotypic differences among individuals are consistent with the interaction of two co-dominant loci, as described in the Methods.

We are focusing our research efforts on two major color pattern loci, *D *and *Cr*, which underlie much of the observed pattern variation in *H. erato*. Both genes are unlinked and alleles at the different loci interact to cause phenotypic shifts across large areas of the wing surface, changing the position, size and shape of red/orange/yellow and melanic patches on both the dorsal and ventral surfaces of the forewings and hindwings. Alleles at the *D *locus primarily act by switching scale color between black (melanin) and red/orange (ommochrome pigments) [12,13]. In contrast, alleles at *Cr *control the positioning of melanin across both the forewing and hindwing, thereby either exposing or covering underlying white and yellow pattern elements (Figure 1). The two loci strongly interact to control the size, shape, and position of both the forewing band and hindwing bar of many races of *H. erato *[9,10,14].

Crossing experiments among the various races of *H. erato *and *H. melpomene *have shown that the genetic basis of the color pattern radiations is similar in these species [15]. In both, a small number of major effect loci, or complex of tightly linked loci, modulate much of the intraspecific pattern variation. Furthermore, the phenotypic effects of many of the major patterning genes are often quite similar between the two species [5,16,17]. For example, *Cr *in *H. erato *and the *N*/*Yb*/*Sb *complex in *H. melpomene *control most of the variation in yellow and white pattern elements in different mimic races of the two species [9,10,17]. Similarly, variation in the major red pattern elements on the forewing and hindwing of *H. erato *and *H. melpomene *can be explained by variation at an unlinked gene, *D*, in *H. erato *and the similarly named *D/B *complex, in *H. melpomene*. In contrast, in *H. melpomene *these switch genes represent clusters of tightly linked elements separated by one cM or less [2,18]. Comparative mapping experiments have shown that the *Yb *complex in *H. melpomene *and the *Cr *locus in *H. erato*, which have analogous phenotypic effects, map to the homologous regions of their respective genomes [15].

There were three primary goals for the study presented here. First, we sought to identify molecular markers linked to the *H. erato *color pattern genes *D *and *Cr*. Second, we used some of these molecular markers to identify and sequence BAC clones containing genomic sequences linked to these color pattern genes. Lastly, we analyzed selected BAC sequences in order to better understand fine-scale characteristics of the *H. erato *genome and to make comparisons with homologous genomic sequences in *H. melpomene *and *B. mori*. Ultimately we found that synteny is highly conserved between *Heliconius *species, and even between *Heliconius *and *B. mori*. We also observed relatively low gene density coupled with a high frequency of novel repeat elements in the *Heliconius *genomic sequences. Together, our data show that comparative genomic analysis between lepidopterans is highly tractable, and that positional cloning of genes underlying color pattern variation in *Heliconius *should be possible using standard methods.

## Results

### Identification of markers tightly linked to color pattern genes

We examined 1440 AFLP *H. erato *polymorphisms using 23 primer combinations (*Eco*CN/*Mse*CNN). The number of AFLP bands per gel ranged between 26 and 132 with a mean of 72 bands per primer combination. Of these, approximately 84% were polymorphic in our outbred F2 cross. The experiment-wide error rate for our screen was approximately 1.0%, as inferred from discrepancies among female informative (FI) markers. In total, we scored 490 Male Informative (MI) and 470 backcross informative (BI) loci. Assuming an estimated *H. erato *genome size of 395 Mb [14], and assuming that AFLP markers are distributed randomly, suggests that we surveyed polymorphisms at approximately 362 kb intervals across the genome. This would suggest a resolution of 1.3 cM assuming that the relationship between physical and recombination distance is 276 kb/cM [9].

Our genome scan identified several AFLP markers 1–3 cM away from *D*. For the other gene, *Cr*, previous work using an identical strategy on crosses of *H. melpomene *provided markers within one cM of this gene in *H. erato *[15]. In total, we identified five AFLP loci within a 3 cM target window around the *D *locus. Across our *H. himera *× *H. erato notabilis *mapping family for *D*, three loci (MI_EcoCA-MseCAA-114 bp, BI_EcoCC-MseCAG-155 bp, BI_EcoCT-MseCCG-139 bp) were perfectly linked and two loci (MI_EcoCC-MseCAC-527 bp, MI_EcoCC-MseCAC-485) showed only one recombinant. We cloned and sequenced several of these AFLP loci and developed PCR primers to amplify them from genomic DNA. Interestingly, two AFLP bands tightly linked to the *D *gene, MI_EcoCC-MseCAC-527 bp, MI_EcoCC-MseCAC-485, were allelic variants of the same locus.

### Identification of BAC clones containing color pattern-linked AFLPs

**Figure 2:**
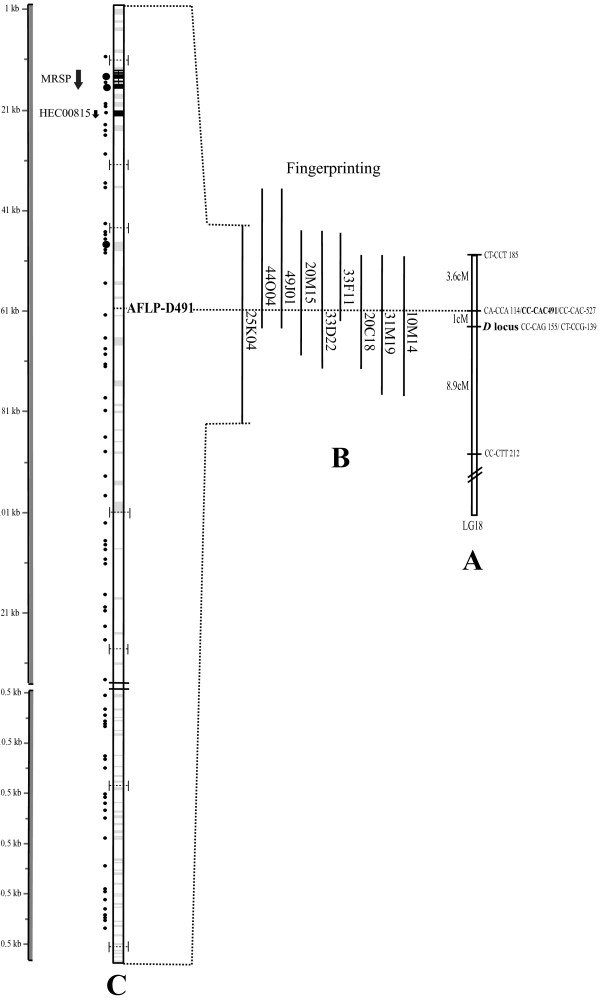
***H. erato *BAC sequence (25_K04) annotation**. Annotation of the BAC sequence (clone BBAM-25K4, accession number AC216670) tightly linked to the *D *color pattern gene. Starting from the right: A) 13.5 cM interval Linkage analysis of LG18, with the gene that control the red pigment (D locus); B) fingerprinting of the positive clones obtained by probing the AFLP CC-CAC-491 (dotted bar); C) sequence analysis of the BAC clone 25_K04, where black circles represent hypothetical ORFs greater than 60 amino acids, with the larger circles representing putative ORFs greater than 150 amino acids. Within each bar, the grey areas indicate repetitive sequence with the black regions indicating exon/intron structure of 2 predicted proteins (with arrow indicating direction) showing a high similarity to known proteins in other arthropods. For gene annotations see Table 2.

**Figure 3:**
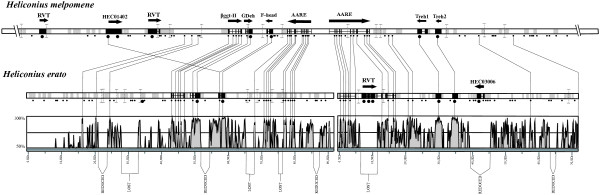
**Fine-scale synteny and sequence conservation between *H. erato *and *H. melpomene***. Top bar represents approximately 180 kb of sequence covering two BACs (clone AEHM-41C10 accession number CR974474; and clone AEHM-7G12 accession number CT955980) for *H. melpomene *and the bottom bar represents 180 kb of sequence in two large contigs for *H. erato *(clone BBAM-38A20, accession numbers AC193804). Black circles below bars represent hypothetical open reading frames (ORFs) greater than 60 amino acids, with the larger circles representing putative ORFs greater than 150 amino acids. Within each bar, the grey areas indicate repetitive sequence with the black regions indicating exon/intron structure of 13 predicted proteins (with arrow above bar indicating direction) showing a high similarity to known proteins in other arthropods. A visual representation of the global alignment between the two genomic sequences and the level of synteny is show at the bottom of the figure. The lines between the two sequences unite regions with high sequence identity (>85% of similarity). For gene annotations see Table 2.

We screened the *H. erato *BAC library with *D*-linked (MI_EcoCC-MseCAC-527 bp) and *Cr*-linked ("*βggt-II" *– *Rab geranylgeranyl transferase beta subunit, βggt-II *gene [15]) probes. In our screens with these and other probes (15 probes in total, with an average of 12 positives per probe), we consistently observed between 9 and 15 PCR-confirmed positives per probe, suggesting the *H. erato *BAC libraries have approximately 10× genome coverage. The largest clones identified from the *D*-linked and *Cr*-linked probing experiments were sequenced at 8× coverage. The *D*-linked clone (BBAM-25K4) was composed of two large sequences that could easily be orientated to produce an approximately 180 kb genomic fragment (Figure 2). Similarly, sequence of the *Cr*-linked clone (BBAM-38A20) was composed of two large sequences that together spanned approximately 165 kb (Figure 3). The probe sequences were clearly identifiable in the *D*-linked and *Cr*-linked BACs and linkage to color pattern genes was confirmed by mapping (see below).

### Chromosome walk in the *Cr *region

**Figure 4:**
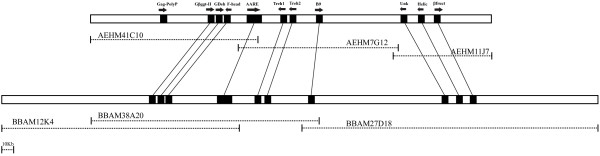
**Gene conservation between *H. erato *and *H. melpomene *contigs**. Comparison of homologous genomic regions linked to the *Cr *color pattern gene in *H. erato *and *Yb *color pattern gene in *H. melpomene*. The top bar represents approximately 280 kb of sequence covering three BACs (clone AEHM-41C10 accession number CR974474, clone AEHM-7G12 accession number CT955980, and clone AEHM-11J7 accession number CU367882) for *H. melpomene*. The bottom bar represents approximately 350 kb of sequence covering three BACs (BBAM-12K4 accession number AC220074, BBAM-38A20 accession number AC193804, and BBAM-27D18 accession number AC199750). Genes showing a high similarity to know proteins in other arthropods are represented with black squares and arrows indicating orientation. The lines between the two sequences unite the homologous genes giving a visual representation of the overall synteny between the two species.

From the sequence of the first *Cr*-linked BAC, identified with the *βggt-II *gene, we designed additional probes to use for a second round of BAC library screening. Specifically we generated two more probes, corresponding to the genes *Trehalase1 *and *B9*, to expand our walk on both directions. With this strategy we identified new BACs on the 3' end that were positive for *B9 *and negative for *Trehalase1 *and others on the 5' end positive for *βggt-II *and negative for *Trehalase1*. After fingerprinting we selected one BAC to sequence from each end. Ultimately, BBAM-27D18 extended the overall contig by 211 kb on the 3' end, while BBAM-12K4 extended the contig on the 5' for 55 kb (Figure 4).

### High frequency of novel repetitive elements in the *Heliconius *genome

The *D- *and *Cr*-linked BAC sequences were AT-rich (65% and 66% AT content, respectively) and contained many repeats (Table 1). Although all three major classes of transposable elements (DNA transposons, LTR, and non-LTR retrotransposons) were present in the genomic sequences (Table 1A), the vast majority of repetitive sequences showed no significant BLAST similarity (*i.e*. e-value < 0.001) to any of the insect genomes currently available NCBI databases nor to any arthropod transposable elements listed in RepBase [19]. *Heliconius*-specific repetitive sequences corresponded to the nine core motifs identified with RepeatFinder (Table 1B; Additional file 1: Novel repetitive elements in *Heliconius *(row sequences)). Of the nine motifs, six are present in both *H. erato *and *H. melpomene*, two are unique to *H. erato*, and one is unique to *H. melpomene *(Table 1; Additional file 1: Novel repetitive elements in *Heliconius *(row sequences)).

**Table 1:** (A, B) – *Heliconius *repetitive elements.

A.																		
**Locus name (origin)**	MARINER_HC (*Hyalophora cecropia*)		MARI_BM (*Bombyx mori*)		GYPSY70-I_AG (*Anopheles gambiae*)		Sake_BM (*Bombyx mori*)		DOC5_DM (*D. melanogaster*)		G4_DM (*D. melanogaster*)		ZENON_BM (*Bombyx mori*)					
**Element Family**	Mariner		Mariner		Gypsy		Daphne		Jockey		Jockey		CR1					
**Element type**	DNA transposon		DNA transposon		LTR retroposon		LTR retroposon		Non-LTR retroposon		Non-LTR retroposon		Non-LTR retroposon					
**Described length**	1255		1310		4858		5140		2791		3856		2599					
											
	era	mel	era	mel	era	mel	era	mel	era	mel	era	mel	era	mel				
				
# regions masked	3	NA	3	NA	NA	1	2	1	5	NA	1	NA	5	1				
Avg length of masked region (+/- Std.Dev.)	85.33 (51.03)	NA	159.33 (28.11)	NA	NA	2062	253 (173.95)	73	440 (275.77)	NA	1243	NA	520.8 (618.74)	44				
Min length of masked region	46	NA	127	NA	NA	NA	46	NA	245	NA	NA	NA	94	NA				
Max length of masked region	143	NA	178	NA	NA	NA	143	NA	635	NA	NA	NA	1574	NA				
Total nucleotide masked	256	NA	478	NA	NA	2062	256	73	880	NA	1243	NA	2604	44				
Proportion of BACs masked	0.07%	NA	0.14%	NA	NA	1.05%	0.07%	0.04%	0.25%	NA	0.35%	NA	0.74%	0.02%				
	
B.																		
**Core Motif**	1		2		3		4		5		6		7		8		8	
									
	era	mel	era	mel	era	mel	era	mel	era	mel	era	mel	era	mel	era	mel	era	mel

Length of core motif	469	NA	460	598	428	NA	384	NA	314	NA	259	NA	266	NA*	147	NA	NA	306
# regions masked	16	2	7	11	29	1	5	NA	11	NA	17	4	310	129	40	9	NA	10
Mean length of masked region (Std.Dev.)	225. (121.71)	70.5 (16.26)	415 (118.3)	406.27 (302.12)	166.69 (127.26)	119 (NA)	341.8 (100.7)	NA	189.36 (90.64)	NA	125.59 (92.66)	76.75 (38.87)	155.25 (70.85)	129.95 (64.26)	89.1 (27.99)	80.11 (21.98)	NA	244.1 (96.74)
Minimum length of masked region	69	59	147	29	37	NA	162	NA	38	NA	28	55	38	40	38	55	NA	46
Maximum length of masked region	439	82	470	740	449	NA	394	NA	314	NA	272	135	287	280	148	116	NA	314
Total nucleotide masked	3603	141	2905	4469	4834	119	1709	NA	2083	NA	2135	307	48126	16763	3564	721	NA	2441
Amount of BACs masked	1.0%	0.1%	0.8%	2.3%	1.4%	0.1%	0.5%	NA	0.6%	NA	0.6%	0.2%	13.6%	8.5%	1.0%	0.4%	NA	1.2%

Summary of identification and repeat masking of repetitive elements in BAC sequences from *H. erato *(accession numbers: AC193804, AC216670) and *H. melpomene *(accession numbers: CR974474, CT955980). A) Repetitive elements identified via similarity to previously described sequences. B) Novel repetitive elements unique to *Heliconius *identified *de novo *using the RepeatMasker software (additional file 1: Novel repetitive elements in *Heliconius *(row sequences)). Each *core motif *is a summary sequence representing a different and unique set of related repeat sequences. Motifs four and five were not observed in *H. melpomene*; Motif nine was not oberseved in *H. erato*. Core Motifs 1,2,3,4,5,6,7 and 8 were identified by RepeatFinder only in *H. erato *but masked by RepeatMasker in *H. melpomene*. (* RepeatFinder identified fragments of this motif in *H. melpomene*, but the groups did not overlap to give a core motif comparable to that in *H. erato*).

### Gene density in *Heliconius *BAC sequences

Gene density appeared to be relatively low across both the *Cr- *and the *D*-linked genomic regions. Although there were a moderate number of predicted open reading frames (ORFs) over 60 amino acids long (Figures 2 and 3), few showed any similarity to known proteins or lepidopteran ESTs, including our own collection of nearly 20,000 *Heliconius *ESTs (Table 2). For example, across the *D*-linked BAC we identified 75 hypothetical proteins using the Kaikogaas annotation tool and our own BLAST analysis. Over 90%, however, were less than 150 amino acids in length, only one of which showed similarity to any known or predicted protein. Across the entire ~190 kb region near the *D *locus in *H. erato *there were only two hypothetical proteins that showed significant homology to a known protein or contained a known structural element. One was similar to a sequence in our EST collection (HEC00815), while the other showed strong homology to a lepidopteran methionine-rich larval storage protein (Table 2). Although there was a smaller absolute number of predicted proteins across the *Cr*-linked BAC, there were more than twice as many predicted proteins of amino-acid length greater than 150 across, all but one of which showed strong homology to known proteins or to *Heliconius *ESTs (Table 2, Figure 2). Overall, the gene density we observed in *H. erato *is similar to what has been observed in the repeat-rich heterochromatin domains of *Drosophila melanogaster*, which averages 2.9 genes per 100 kb (versus 12.6 genes per 100 kb in euchromatin) [20].

**Table 2:** BAC annotation summary.

Predicted gene	BAC Accession number			Organism	Identities	E value
		
	H. erato	H. melpomene	B. mori			
Putative reverse transcriptase (RVT)	NA	CR974474	2529(818322–834985;7e-13)*	Medicago truncatula	48%	e-140
HEC01402 (DT665615)	NA	CR974474	2795(2232547–2233374;9e-26)*	Heliconius erato	92%	0.0
Putative reverse transcriptase (RVT)	NA	CR974474	2136(919260–923481;1e-10)*	Aedes aegypti	29%	5e-76
Galactokinase	AC193804/AC220074	NA	2829(2954764–2958794;5e-43)*	Xenopus laevis	42%	1e-61
Rab geranylgeranyl transferase b subunit (βggt-II)	AC193804/AC220074	CR974474	2829(2933018–2938173;e-129)*	Danio rerio	68%	1e-98
Glucose dehydrogenase (GDeh)	AC193804/AC220074	CR974474	2829(2929161–2931568;e-111)*	Aedes aegypti	36%	3e-91
Forkhead box J1 (F-head)	AC193804/AC220074	CR974474	2829(2921212–2923967;e-123)*	Tribolium castaneum	43%	3e-57
Acylamino-acid-releasing enzyme (AARE)	AC193804/AC220074	CR974474	2829(2895678–2909890;e-125)*	Tribolium castaneum	39%	2e-84
Putative reverse transcriptase (RVT)	AC193804	NA	3058(6735155–6740613;e-100)*	Drosophila simulans	42%	0.0
Acylamino-acid-releasing enzyme (AARE)	AC193804	CR974474/CT955980	2829(2910836–2912522;3e-65)*	Tribolium castaneum	50%	2e-48
Trehalase (Treh1)	AC193804	CT955980	2829(2866386–2868122;0.0)*	Bombyx mori	50%	e-158
Trehalase (Treh2)	AC193804	CT955980	2829(2861182–2862921;0.0)*	Bombyx mori	60%	0.0
HEC03006 (DT668569)	AC193804	NA	3058(4660174–4667549;2e-08)	Heliconius erato	96%	0.0
Methionine-rich storage protein 2 (MRSP)	AC216670	NA	3026(2396448–2401216;0.0)*	Manduca sexta	66%	0.0
HEC00815 (DT661873)	AC216670	NA	NA	Heliconius erato	96%	0.0
B9	AC199750	CT955980	2829(2830788–2833491;3e-79)*	Danio rerio	39%	3e-33
Unkempt (Unk)	AC199750	CU367882	2829(2748736–2755694;e-110)*	Aedes aegypti	76%	e-102
Putative DNA helicase recQ (Helic)	AC199750	CU367882	2829(2870771–2872660;0.0)*	Apis mellifera	51%	e-173
Beta fructosidase FruA (βfruct)	AC199750	CU367882	2829(2704806–2706326;e-176)*	Apis mellifera	36%	7e-74

Annotation summary for four *H. erato *BACs (accession numbers: AC193804, AC216670, 220074, 199750). Predicted gene function and BLASTp data (species hit, % identity, and e-value) are reported, as are accession numbers of the *H. erato, H. melpomene*, and *B. mori *genomic sequences containing the predicted genes. (* scaffold ID, position and e value as seen in the silkworm genome database: SilkDB)

### Fine-scale microsynteny between *H. erato *and *H. melpomene *genomic sequences

VISTA analysis [21] (70% identity, 30 bp window – see Methods) showed a 35% conservation between the 164 kb *H. erato Cr*-linked BAC clone (BBAM-38A20) and the homologous sequence from *H. melpomene *(Figure 3). All of the predicted genes showed strong similarity (80–95% identity) between the two species and perfect overall synteny (Figure 3 and 4). Furthermore, a significant portion of 57 kb of presumed non-coding sequence (*i.e*. did not show notable open reading frames) was highly conserved between the two species (Figure 3). Despite the overall conservation between *H. erato *and *H. melpomene *sequences, two ESTs did show a difference between the species. Firstly, HEC03006 was found only in *H. erato*, and corresponded to a large indel sequence. Also, HEC01402 was found in the *H. melpomene *BAC sequence but not the *H. erato *sequence, although it shared some similarity with an exon of the *Forkhead *gene in the *H. erato *BAC sequence. For HEC01402, it is likely that the *H. erato *BAC sequence did not extend far enough to cover the homologus genomic region containing the gene in *H. melpomene*.

### Conservation of gene order between *H. erato *and *B. mori*

**Figure 5:**
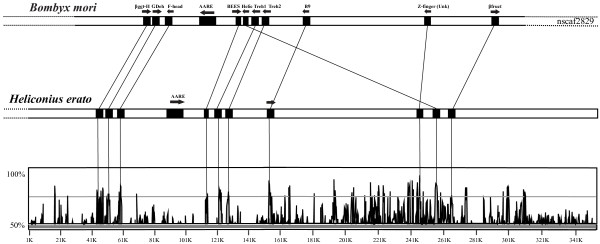
**Conservation of gene order and distances between *H. erato *and *B. mori***. VISTA analysis shows sequence conservation between coding regions in the *Cr*-linked *H. erato *BAC clones (BBAM-38A20, accession number AC193804; BBAM-27D18, accession number AC199750 and BBAM-12K4, accession number AC220074) and the *B. mori *scaffold sequence 2829 (position: 2706326–2993165). Genes showing a high similarity to know proteins in other arthropods are represented with black squares and arrows indicating orientation (when orientation is different arrows are displayed for both species). The lines between the two sequences unite the homologous genes giving a visual representation of the overall synteny between the two species.

We found evidence for fine-scale synteny between *H. erato *and *B. mori *in the 420 kb genomic region linked to the *Cr *color pattern gene (Figure 5). No evidence for microsynteny was observed between *D*-linked *H. erato *genomic regions and *B. mori*, due to the lack of conserved genes in the *D*-linked clone. *B. mori *scaffold sequence (nscaf2829), downloaded from SilkDB [22], contained all of the major genes annotated on the *Cr*-linked BAC clones (Table 2, Figure 5). All genes were unambiguously identified: *βggt-II *(nscaf2829, position 2933018–2938173); *Glucose dehydrogenase *(nscaf2829, position 2929161–2931568); *Forkhead Box *(nscaf2829, position 2921212–2923967); *Trehalase1 *(nscaf2829, position 2866386–2868122); *Trehalase2 *(nscaf2829, position 2861182–2862921); *B9 *(nscaf2829, position 2830788–2833491); *Unkempt *(nscaf2829, position 2748736–2755694); *Beta fructosidase FruA *(nscaf2829, position 2704806–2706326). With the exception of the *DNA helicase *(nscaf2829, position 2870771–2872660), which appears to have been translocated, all of the gene orders and distances in *Heliconius *and *B. mori *are highly conserved. For example, *Glucose dehydrogenase *and *Forkhead Box *were separated by 5300 bp in *H. erato *and 5193 bp in *B. mori*, while *Trehalase1 *and *Trehalase2 *were separated by 3110 bp in *H. erato *and 3464 bp in *B. mori*. All seven genes showed 70–85% nucleotide acid sequence similarity between species (Figure 5). In addition to the major genes, there were many other genomic regions between *Heliconius *and *B. mori *with a nucleotide acid sequence similarity higher than 85% that did not show BLAST similarities to any known proteins.

## Discussion

### High levels of fine-scale genomic conservation between *Heliconius *species

We have previously demonstrated that the *Cr *locus in *H. erato *and the *Yb *gene of *H. melpomene *map to homologous areas of the genome [15]. BAC genome sequence data for a region tightly linked to the *Yb *gene was obtained from *H. melpomene *using methodology similar to that described here. A single gene marker developed from the *H. melpomene *sequence mapped close to the *Cr *locus in *H. erato*. Here we provide the first genomic sequence evidence that, across a broad region around this gene, gene order and gene content is conserved. Across a 420 kb overlapping region all putative proteins showing high similarity to known proteins were in the same order in the two co-mimics (Figure 3 and 4). This further supports the hypothesis that a homologous gene, or set of genes, is responsible for color pattern variation in the two species. Indeed, with the exception of two large ORFs with strong sequence similarity to a reverse transcriptase (Table 2, Figure 3), gene order in the Cr-linked *H. erato *region and the *N*/*Yb*/*Sb*-linked *H. melpomene *region was nearly perfectly preserved. Furthermore, many of the smaller ORFs, as well as some non-coding sequence, were highly conserved both in the relative order and sequence. Generally, the *H. erato *and *H. melpomene *genomes appear to be structurally very similar. Because of this, linkage analyses and positional cloning efforts in each individual species should be highly informative for the co-mimetic species, and probably for the genus as a whole.

### The difference in *H. erato *and *H. melpomene *genome sizes

One of the most obvious differences between the *H. erato *and *H. melpomene *genomic sequences was the larger physical distance between homologous anchor points in *H. erato *relative to *H. melpomene*. This observation was probably related to the fact that the genome of *H. erato *is about 30% larger than that of *H. melpomene *[9,10]. In this respect, it was notable that the difference in genome sizes between the two species was roughly proportional to the size of a number of sequence blocks that are absent in *H. melpomene *relative to *H. erato *in our genomic sequences (Figure 3). Many of these blocks were comprised of *Heliconius*-specific repetitive sequences, or showed strong similarity to known mobile genetic elements. These indel blocks appeared to be primarily noncoding sequences because none of them contained protein-coding sequences, with the exception of one EST (HEC03006) and a *reverse transcriptase*.

Differences in the genome size between closely related species is common and is usually associated with differences in abundance of different classes of noncoding DNA [23]. It has been suggested that the insertion and replication of repetitive elements, as well as indel biases, can lead to profound differences in genome size [24,25]. Our observations suggest that both of these effects might be relevant in *Heliconius*, however, the differences in amount of repetitive DNA sequences in indels, at least over the small region that we examined, were not large enough to completely account for the differences in the genome size of the two species.

### Novel repetitive elements in *Heliconius*

Using RepeatFinder [26] and RepeatMasker [27], we identified 16 different repeated elements in the *Heliconius *genomic sequences (Table 1). In total, repetitive sequences accounted for about 20% of the *H. melpomene *and about 26% of the *H. erato *genomic sequence. Seven of these repeated elements corresponded to previously described sequences (Table 1A). Because we were unable to detect any of the remaining nine repeats (all identified via RepeatFinder) in public sequence databases we assume these nine repeats represent novel repetitive elements unique to the *Heliconius *genus (Table 1B, Additional file 1: Novel repetitive elements in *Heliconius *(row sequences)). The seven previously described elements were larger (~1–5 kb) relative to the nine novel elements (100–600 bp) and occurred much less frequently. Most instances of these novel repeats observed in the BAC sequences were intact, full-length, highly similar versions of the core motifs. However, a wide range of fragmentation and divergence relative to the core motifs was also observed among repetitive regions. Motif #7 in *H. erato *best exemplifies this pattern, as it was the most abundant repeat observed, with 310 BAC regions showing significant similarity to the 266 bp core motif. These regions ranged continuously in size from 37 to 286 bp and in divergence from 2% to 32%. All other motifs showed a qualitatively similar pattern where BAC regions corresponding to a novel element ranged from being highly similar to the core motif sequence to being fragmented and divergent. This pattern is consistent with a process of motif replication and insertion followed by mutational degradation, suggesting these novel repeat motifs likely represent some sort of transposable element such as short interspersed nuclear elements (SINEs) or miniature inverted-repeat terminal elements (MITEs) [28]. Both SINEs and MITEs have been reported from Lepidoptera [29,30]. More work is required to confidently determine the origin of these novel repetitive sequences in *Heliconius*. We are hopeful that future genomic sequences from other butterfly species will allow a better understanding the phylogenetic distribution and evolutionary origins of some of the observed repeats.

### Preliminary evidence for fine-scale synteny between *Heliconius *and *Bombyx*

The observed fine scale synteny within *Heliconius *complements a recent study showing that patterns of macrosynteny are strongly conserved across the Macrolepidoptera [11]. This previous study demonstrated that a total of 70 markers mapped in *H. melpomene *and *B. mori *showed large-scale patterns of synteny across the genome (taking into account a number of putative chromosomal fusions that explain the difference in chromosome number between these species). Our data further suggests that synteny has been preserved between *Heliconius *and *B. mori *on a much finer scale. Specifically, we show here that seven predicted genes in the *Cr *region have a similar order in the homologous *B. mori *genomic sequence (Figure 5). Although this is a very small sampling of the genome as a whole, it is still notable that gene order, as well as intergenic distances, has been preserved over such a long time scale.

### Chromosome walking towards *Heliconius *color pattern genes

The goal of this study was to identify and characterize regions of the genome linked to wing pattern polymorphism in *Heliconius *butterflies. We did not necessarily expect these initial BAC sequences to contain the color pattern genes themselves, however, these sequences provide important genomic "anchors" for ongoing positional cloning work. Fine-scale mapping experiments imply that we are very near the *D *and *Cr *color pattern loci. A microsatellite marker at the 5' end of the *D*-linked BAC showed 7 recombinants across 444 individuals, suggesting that this end is about 1.9 cM from the gene. There were two fewer recombinants for a marker developed from exon sequence of the *Methionine Rich Storage Protein *(*MRSP*) gene at the 3' end of the BAC. This marker is about 150 kb from our 5' microsatellite marker, suggesting that *D *is a further 1 cM in the 3' direction. We appear to have come down even closer to the *Cr *gene. In this case, a marker developed from the *βggt-II *gene near the 5' of the BBAM-38A20 BAC clone showed no recombinants across 430 individuals. A similar result was obtained with a marker developed from the *Putative DNA helicase Rec-Q *in the middle of the BBAM-27D18 BAC clone, suggesting that the zero recombinant interval might be somewhat large. For this reason, given an expected relationship of physical to recombination distance of 275 kb/cM [9], an expectation consistent with our initial mapping results, we are optimistic to identify the genomic recombinant interval containing both genes with the next one (*Cr*) to three (*D*) BAC steps.

In terms of identifying color pattern genes, there are obvious practical benefits of a fine-scale preservation of gene order between species. Foremost, conservation should greatly facilitate the identification of functional genes and the discovery of some regulatory elements associated with these genes [31,32]. Indeed, there were numerous conserved regions that were not simply protein coding regions (Figure 3). Even though this analysis covers only a small portion of the *Heliconius *genome, it confirms, for the first time, at a fine scale level what has been seen in comparative mapping projects concerning gene order conservation between different species in the genus [9,10,33].

A comparative approach will be particularly important for pinpointing the regions responsible for pattern variation in *Heliconius*. Pattern formation in *Heliconius *probably involves discrete changes in conserved protein coding or regulatory regions [2,12]. There is little precedent for what to expect, however, variation in pattern formation could be controlled by a number of *cis*-regulatory elements of a single gene, clusters of duplicated genes with divergent function, or clusters of non-paralogous but functionally-related genes.

## Conclusion

The mimetic wing patterns of *Heliconius *stand out as one of the best examples of an adaptive radiation. We are using a strategy that couples growing genomic resources with high-resolution linkage analysis in order to gain a fuller appreciation of the genetic basis of this radiation. We have identified regions of the *Heliconius *genome tightly linked to genes that modulate pattern variation and, for one of these regions, we have demonstrated the fine-scale preservation of gene order between distantly-related *Heliconius *species and across Lepidoptera (*Heliconius *and *B. mori*). This conservation is significant because it will greatly facilitate efforts for gene identification through parallel and complementary efforts in different species. It is our hope that further fine-scale mapping, complemented by targeted genomic sequencing, will allow us to identify the genes that underlie wing pattern variation and diversity in the genus *Heliconius*.

## Methods

### Cross strategy

We generated large F2 mapping families by crossing two different geographic races of *H. erato *to the same stock of *H. himera *(Figure 1). We followed segregating variation at both the *D *and the *Cr *loci in crosses between *H. erato cyrbia *and *H. himera *and the *D *and *Sd *loci using crosses between *H. erato notabilis *and *H. himera *(Figure 1). All crosses were carried out in the *Heliconius *insectary at the University of Puerto Rico from stocks originally gathered from the wild: *H. himera *was collected in Vilcabamba, Ecuador (79.13 W, 4.6 S), *H. erato notabilis *was collected from an area near Puyo, Ecuador (78.0 W, 1.5 S), and *H. erato cyrbia *was collected near Guayquichuma Glen, Ecuador (79.6 W, 3.9 S). After eclosion butterflies were euthanized, their wings removed for later morphological analysis, and their bodies frozen at -80 °C for later molecular analysis.

### Identifying AFLP markers linked to color pattern genes

Genomic DNA was extracted following procedures described in [14] and approximately 500 ng of DNA was used as template of our AFLP reactions. Genomic DNA restriction digestions and the ligation of oligonucleotide adapters were performed using the Core Reagent Kit (Invitrogen Life Technologies) following manufacture's instructions. For all the other steps of the AFLP analysis (e.g. serial dilutions and PCR amplification protocols), we followed the modifications of the original protocol described by Vos et al. [34] as outlined in Papa et al. [35].

To efficiently identify primer combinations that contained loci tightly linked to color pattern genes, we used a modification of the "bulk segregant analysis" method [36]. Specifically, we screened 23 AFLP primer combinations across an initial panel of 48 individuals arranged by color pattern genotype. Each reaction was run on a 10% polyacrylamide gel and visualized using fluorescently labeled (IRDye 700 or 800) EcoCN primers on a NEN^® ^Global Edition IR2 DNA Analyzer (LI-COR^® ^Biosciences, Lincoln, NE). In our screening gels, individuals with distinctive color-pattern genotypes were grouped and these groups were run side-by-side. For example, in the *H. erato notabilis *× *H. himera *cross we structured the gel with the following four genotypic groups: 1) *D*^*him*^*D*^*not*^*, Sd*^*him*^*Sd*^*him*^; 2) *D*^*him*^*D*^*not*^*, Sd*^*not *^Sd^*not*^; 3) *D*^*him*^*D*^*him*^*, Sd*^*him*^*Sd*^*not*^; 4)*D*^*not*^*D*^*not*^*, Sd*^*him*^*Sd*^*not *^(Figure 1). In this way, we easily identified marker loci linked to the color pattern genes and those primer combinations showing tightly linked markers were further assayed on all 120 individuals in the brood.

Because of the nature of our outbred F2 cross design [9,10] we observed AFLP loci in all possible phases and segregation patterns. For each AFLP primer combination, we scored four different AFLP marker types: (1) monomorphic, (2) female-informative (FI) (AFLP band present in the mother and absent in the father), (3) male-informative markers (MI) (AFLP band present in the father but absent in the mother), and (4) markers that were present in both parents but segregated in offspring (BI). Both MI and FI markers were expected to segregate in a 1:1 ratio, whereas the BI markers, which were heterozygous in the parents, segregate in a 3:1 ratio. There is no crossing over during the oogenesis in Lepidoptera [37,38] and FI markers on the same chromosome are inherited as a linkage block [10]. Thus, only the MI and BI provided information on recombination distance between AFLP polymorphisms and color pattern genes. Nonetheless, the FI markers were extremely useful because they allowed us to unambiguously identify chromosomal linkage groups and estimate background error rate of the AFLP technique [9,10].

Genotypes for the color pattern genes were inferred from wing pattern phenotypes. Based on the amount of red, white and yellow in the forewing and hindwing, nine genotypes were observed among both groups of F2 progeny. For the *D *gene, shared by all three grand-parental types: homozygotes for the *H. himera *allele (*D*^*hi*^*D*^*hi*^) show only red present on the hind-wing bar, when *D *homozygotes for *H. erato cyrbia *or *H. erato notabilis *alleles (*D*^*cyr *^*D*^*cyr*^; *D*^*not*^*D*^*not*^) have red scales on the forewings, while *D *is heterozygotes between *H. himera *and the two *H. erato *races (*D*^*hi *^*D*^*cyr*^; *D*^*hi *^*D*^*not*^) have red pigmented scales on both forewings and hindwings. The *Cr *gene segregates only in the *H. erato cyrbia *× *H. himera *cross, and has an epistatic effect on the *D *gene by modulating the amount of red. Homozygotes with *H. himera *alleles (*Cr*^*hi*^*Cr*^*hi*^) show a typical yellow forewing band when *D *is pure *H. himera *(*Cr*^*hi*^*Cr*^*hi*^; *D*^*hi*^*D*^*hi*^) and a red/white forewing band (total amount of red pigments < 50%) when *D *is either homozygous for *H. erato cyrbia *alleles (*Cr*^*hi*^*Cr*^*hi*^; *D*^*cyr*^*D*^*cyr*^) or heterozygous (*Cr*^*hi*^*Cr*^*hi*^; *D*^*hi*^*D*^*cyr*^). When the *Cr *color pattern gene is heterozygous (*Cr*^*hi*^*Cr*^*cyr*^) a yellow forewing shadow band is present when the *D *gene is homozyous for *H. himera *alleles (*Cr*^*hi*^*Cr*^*cyr*^; *D*^*hi*^*D*^*hi*^), and an almost all red forewing band (red >50%) when *D *homozygote for *H. erato cyrbia *alleles (*Cr*^*hi*^*Cr*^*cyr*^; *D*^*cyr*^*D*^*cyr*^) or heterozygote (*Cr*^*hi*^*Cr*^*cyr*^; *D*^*hi*^*D*^*cyr*^). A white trailing dorsal edge and a yellow bar on the ventral hindwing identify the pure *H. erato cyrbia *form (*Cr*^*cyr*^*Cr*^*cyr*^) with a totally black forewing when *D *is homozygous for *H. himera *alleles (*Cr*^*cyr*^*Cr*^*cyr*^; *D*^*hi*^*D*^*hi*^), or totally red when the *D *gene is pure for *H. erato cyrbia *alleles (*Cr*^*cyr*^*Cr*^*cyr*^; *D*^*cyr*^*D*^*cyr*^) or heterozygous (*Cr*^*cyr*^*Cr*^*cyr*^; *D*^*hi*^*D*^*cyr*^).

To score color patterns, one must also understand activity of the *Sd *gene. *Sd *segregates in the *H. erato notabilis *× *H. himera *cross, where it controls the shape of the melanic window in the middle forewing of *H. himera *and shows a clear interaction with the distal forewing patch of *H. erato notabili*s. In the pure *H. himera *(*Sd*^*hi*^*Sd*^*hi*^) a typical forewing band is shown, while in the pure *H. erato notabilis *(*Sd*^*not*^*Sd*^*not*^) a shortened basal forewing patch, as well as a smaller distal forewing patch are evident. The heterozygotes (*Sd*^*hi*^*Sd*^*not*^) present an intermediate form lacking a distal patch (as found in *H. erato notabilis*) and have a shortened basal patch showing melanin anterior to the costal vein (this area is normally light colored in *H. himera*).

### Isolating and characterizing AFLP markers

We screened all primer combinations that generated BI or MI AFLP markers strongly associated with particular color pattern genes in our initial "bulked" sample across all 120 individuals from our mapping family. We excised and cloned those tightly linked AFLP markers larger than 160 base-pairs using a three-step strategy. First, the band was isolated from a polyacrylamide gel using a LI-COR^® ^Biosciences Odyssey^® ^Infrared Imaging System. We followed methods outlined in [39] and used a grid to position and excise specific fragments with a scalpel. We validated each excision by re-scanning the gel to confirm that we had removed the correct fragment. All gel fragments were placed in 15 ml of 1× TE and frozen at -80°C. Next, we re-amplified the AFLP using the original selective primer combination and the original PCR conditions. We generated template for this reaction by freeze/thawing the excised band three times. In each freeze/thaw cycle, we collected the resulting supernatant after the band was frozen for one hour at -80°C, heated at 55°C for 15 min and centrifuged for 15 min at 15,000 rpm. We checked amplification success by running PCR products on polyacrylamide gels adjacent to the positive control original AFLP reactions. Finally, we cloned the PCR product using Invitrogen's TOPO TA^® ^Cloning kit. PCR amplified inserts from 10–15 positive clones were again verified size on a polyacrylimide gel and those of the correct size were sequenced using DYEnamicT ET Terminator Cycle Sequencing Kit (Amersham Biosciences). Sequencing reactions were run on a MegaBACE 500 (Amersham Bioscience) or a 3130 DNA PRISM (ABI) at the Sequencing Facilities of the University of Puerto Rico, Rio Piedras. Resulting sequences were aligned by eye and PCR primers were designed using OLIGO version 4.0, and tested in genomic extracts from a panel of *H. erato *individuals.

### Probing the *H. erato *BAC library

Two *H. erato *BAC libraries, one partially restricted with *Eco*RI and one with *BamHI*, were created from a line of *H. erato petiverana *collected in Gamboa, Panama and inbred for several generations. The *H. erato *BAC library was constructed by C. Zhang (TAMU) and M. R. Goldsmith (URI) following the procedure outlined in Wu et al. [40]. Both libraries contain 19,200 clones arrayed in 384-well plates and the average insert size for the *Eco*RI and *Bam*HI libraries was estimated to be 153 kb and 175 kb, respectively. Libraries were gridded onto nylon membranes using a strategy where each clone is spotted twice to facilitate the identification of "true" positives. AFLP probes were labeled with P^32 ^using the Prime-It II Random Primer Labeling Kit (Stratagene, CA, USA). The resulting radioactive labeled products were cleaned using a sephadex purification column and hybridized to the filters overnight at 65 degrees in Church Buffer (0.5 M NaHPO4 pH 7.2, 7%SDS, 1 mM EDTA, and 1%BSA) with rotation. The filters were washed twice with 2× SSC +0.1%SDS, then once or twice with 1× SSC + 0.1%SDS. The washed filters were placed on film for 1–5 days at -80 degrees, depending on signal strength.

### BAC fingerprinting, sequencing, and annotation

All positive clones identified in our screen of the *H. erato *BAC libraray were PCR-confirmed using probe specific primers. Clones were then grown overnight on agar plate and single colony was used to inoculate TB media. Insert DNA was extracted using the Qiagen Maxi prep kit. This insertion size of each BAC clone is estimated by summing all fragments after restriction enzyme digestion using *Eco*RI or *Bam*HI. The largest clone identified from our fingerprinting experiments was sequenced and assembled by the Baylor Genomics Center. Clones were first sheared to create 4–6 kb fragments and subcloned into pUC19. Approximately 8× sequence coverage of each BAC was then generated in paired 600–800 bp reads. Data were assembled using PHRAP [41] and edited in a GAP4 [42] database.

The BAC sequences were analyzed using a variety of sequence annotation programs. First we used Kaikogaas [43], an automated annotation package for gene prediction [44], to identify possible genes. Kaikogaas integrates a variety of programs for gene prediction and structural analysis of genomic sequence including software for coding region and splice-site prediction, sequence homology analysis, protein localization site prediction, and protein classification and secondary structure prediction. All putative open reading frames were also compared directly to our database of *Heliconius *Expressed Sequence Tags (ESTs) located in ButterflyBase [45] using BLAST [46]. Any EST that showed highly significant similarity (e-value of ≥ 10^-40^) to a putative ORF was then directly compared to the full genomic sequence. In this way, we could better assess possible homology versus similarity due to repetitive DNA elements (see below). To identify the *B. mori *sequences homologous to the *H. erato *BAC sequences, we performed a tBLASTn against the whole-genome shotgun reads of *B. mori *using as a query the translated protein sequence predicted by Kaikogaas.

### Comparative sequence analysis

A linkage strategy similar to ours was previously used to identify BACs near the *Yb *gene complex in *H. melpomene *[15]. Sequencing and finishing of three contiguous *H. melpomene *BACs across this region was carried out by the Wellcome Trust Sanger Institute (accession numbers: CR974474, CT955980, CU367882). We used VISTA [21] to identify similarities between *H. erato*, *H. melpomene*, and *B. mori *genomic sequences. Pairwise genomic alignments were performed on the mVISTA server using the Avid alignment algorithm and the results were displayed together with the position of the annotations (ORFs, genes, mobile DNA, microsatellites). Each annotation or new genomic region identified from the comparative analysis that showed a strong similarity (≥ 80% conserved) was verified by aligning the sequences from both species using the LAGAN program [47] to get an accurate DNA sequence assembly.

We also searched the *H. erato *and *H. melpomene *BAC sequences for novel repetitive sequence as well as previously described transposable elements. To identify novel repetitive sequences in the BACs, we used the RepeatFinder software [42,48]. RepeatFinder is explicitly designed to find complex repeated motifs in large contiguous blocks genomic DNA sequence. Its algorithm uses BLAST to iteratively query segments of the input sequence against the intact input sequence. It then combines subsequences with high BLAST similarity into groups, which are further refined by considering the variability in length and divergence among constituent subsequences. The final output is a list of groups of aligned, highly similar subsequences. It is important to note that because the algorithm uses local alignments (generated by BLAST), different groups may overlap in part or in whole. This overlap allows the groups to be clustered into just a few contigs representing a set of "core motifs" to which each group can be uniquely assigned. Thus, each core motif is a consensus of consenses.

We submitted the concatenated BAC sequences from each species to RepeatFinder using default parameters except for the following: Block Size = 2000, Minimum Repeat Size = 20, Maximum Repeat Size = 700. Larger values for the Maximum Repeat Size parameter were tried, but no groups larger than ~600 bp were ever identified. Core motifs were assembled from group consensus sequences >35 bp in length using the default parameters in CodonCode Aligner software (CodonCode Corp., Dedham, MA). For both species' sets of core motifs, all-vs-all BLASTn searches were performed 1) between species to identify motifs shared between species and 2) within species to verify that motifs within species were unrelated. BLASTn was also used to search two additional databases for sequences similar to the core motifs. First we searched a combined database of the completed genomic or whole genome shotgun sequences from all 20 insect genome projects currently available at NCBI. We also searched all arthropod transposable elements available in the Repbase library of transposable elements [19].

We simultaneously identified and masked BAC regions corresponding to both the novel core motifs and known repetitive sequences using RepeatMasker [27]. We combined core motif sequences with sequences of all arthropod transposable elements from RepBase into a single database. We queried this database with the concatenated BAC sequences using RepeatMasker and the CrossMatch search algorithm on the 'slow' setting to maximize sensitivity. The resulting data was summarized using custom scripts implemented in the R statistics package [49].

## Authors' contributions

RP assisted in project design, mapping, marker isolation, sequence analysis, figure preparation, and writing manuscript. CMM and GH assisted in screening BAC clones. JRW and BAC assisted with sequence analysis and writing the manuscript. RC assisted in BAC sequencing and assembly of *Heliconius erato*. R-fC and NC assisted in BAC sequencing and assembly of *Heliconius melpomene*. DDK assisted in crossing and design of AFLP bulk strategy, AFLP mapping, figure preparation, and commenting on the manuscript. CDJ and LF provided *H. melpomene *data and commented on the manuscript. RDR assisted in marker isolation, sequence analysis, figure preparation, and writing the manuscript. WOM assisted with project design, crossing and mapping, sequence analysis, and writing the manuscript.

## Supplementary Material

Additional File 1 Sequences of the *Heliconius *novel repetitive elements. Core Motif sequences of the nine novel *Heliconius *repetitive elements identified with RepeatFinder in BAC sequences from *H. erato *(accession numbers: AC193804, AC216670) and *H. melpomene *(accession numbers: CR974474, CT955980). Click here for file

## References

[B1] McMillan WO, Monteiro A, Kapan DD (2002). Development and evolution on the wing. Trends Ecol Evol.

[B2] Joron M, Jiggins CD, Papanicolaou A, McMillan WO (2006). *Heliconius *wing patterns: an evo-devo model for understanding phenotypic diversity. Heredity.

[B3] Brown KS (1981). The biology of *Heliconius *and related genera. Annu Rev Entomol.

[B4] Jiggins CD, Naisbit RE, Coe RL, Mallet J (2001). Reproductive isolation caused by colour pattern mimicry. Nature.

[B5] Sheppard PM, Turner JRG, Brown KS, Benson WW, Singer MC (1985). Genetics and the evolution of Müllerian mimicry in *Heliconius *butterflies. Philos Trans R Soc Lond B Biol Sci.

[B6] Brower AVZ (1994). Phylogeny of *Heliconius *butterflies inferred from mitochondrial DNA sequences (Lepidoptera: Nymphalidae). Mol Phylogenet Evol.

[B7] Beltrán M, Jiggins CD, Brower AVZ, Bermingham E, Mallet J (2007). Do pollen feeding, pupal-mating and larval gregariousness have a single origin in *Heliconius *butterflies? Inferences from multilocus DNA sequence data. Biol J Linn Soc Lond.

[B8] Jiggins CD, McMillan WO (1997). The genetic basis of an adaptive radiation: warning colour in two *Heliconius *species. Proc R Soc Lond B Biol Sci.

[B9] Kapan DD, Flanagan NS, Tobler A, Papa R, Reed RD, Acevedo Gonzalez J, Ramirez Restrepo M, Martinez L, Maldonado K, Ritschoff C (2006). Localization of Mullerian mimicry genes on a dense linkage map of *Heliconius erato*. Genetics.

[B10] Jiggins CD, Mavarez J, Beltrán M, McMillan WO, Johnson S, Birmingham E (2005). A genetic linkage map of the mimetic butterfly, *Heliconius melpomene*. Genetics.

[B11] Pringle EG, Baxter SW, Webster CL, Papanicolaou A, Lee SF, Jiggins CD (2007). Synteny and chromosome evolution in the lepidoptera: Evidence from mapping in *Heliconius melpomene*. Genetics.

[B12] Reed RD, McMillan WO, Nagy LM (2008). Gene expression underlying adaptive variation in *Heliconius *wing patterns: non-modular regulation of overlapping cinnabar and vermilion prepatterns. Proc R Soc B Biol Sci.

[B13] Gilbert LE, Forrest HS, Schultz TD, Harvey DJ (1988). Correlations of ultrastructural and pigmentation suggest how genes control development of wing scales on *Heliconius *butterflies. J Res Lep.

[B14] Tobler A, Kapan DD, Flanagan N, Johnson S, Heckel D, Jiggins CD, McMillan WO (2005). First generation linkage map of *H. erato*. Heredity.

[B15] Joron M, Papa R, Beltrán M, Chamberlain N, Mavárez J, Baxter S, Abanto M, Bermingham E, Humphray SJ, Rogers J (2006). A conserved supergene locus controls colour pattern diversity in *Heliconius *butterflies. PLOS Biology.

[B16] Turner JRG, Crane J (1962). The genetics of some polymorphic forms of the butterflies *Heliconius melpomene *Linneaus and *H. erato *Linneaus, I: major genes. Zoologica.

[B17] Mallet J (1989). The genetics of warning colour in Peruvian hybrid zones of *Heliconius erato *and *H. melpomene*. Proc R Soc Lond B Biol Sci.

[B18] Baxter SW, Papa R, Chamberlain N, Humphray SJ, Joron M, french-Constant R, McMillan WO, Jiggins CD (2008). Parallel evolution in the genetic basis of Müllerian mimicry in *Heliconius *butterflies. Genetics.

[B19] Jurka J, Kapitonov V, Pavlicek A, Klonowski P, Kohany O, Walichiewicz J (2005). Repbase Uptade, a database of eukaryotic repetitive elements. Cytogenet Genome Res.

[B20] Smith CD, Shu SQ, Mungall CJ, Karpen GH (2007). The Release 5.1 annotation of *Drosophila melanogaster *heterochromatin. Science.

[B21] Frazer KA, Pachter L, Poliakov A, Rubin EM, Dubchack I (2004). VISTA: computational tools for comparative genomics. Nucleic Acids Res.

[B22] Silkworm Genome Database: SilkDB. http://silkworm.genomics.org.cn/.

[B23] Comeron MJ (2001). What controls the length of noncoding DNA?. Curr Opin Genet Dev.

[B24] Petrov DA (2002). DNA loss and evolution of genome size in *Drosophila*. Genetica.

[B25] Imai S, Sasaki T, Shimizu A, Asakawa S, Hori H, Shimizu N (2007). The genome size evolution of medaka (*Oryzias latipes*) and fugu (*Takifugu rubripes*). Genes Genet Syst.

[B26] RepeatFinder@BioHPC. http://cbsuapps.tc.cornell.edu/repeatfinder.aspx.

[B27] RepeatMasker. http://repeatmasker.org/.

[B28] Kramerov DA, Vassetzky NS (2005). Short retroposons in eukaryotic genomes. Int Rev Cytol.

[B29] Yang CS, Teng XY, Zurovec M, Scheller K, Sehnal F (1998). Characterization of the P25 silk gene and associated insertion elements in *Galleria mellonella*. Gene.

[B30] Kumaresan G, Mathavan S (2004). Molecular diversity and phylogenetic analysis of mariner-like transposons in the genome of the silkworm *Bombyx mori*. Insect Mol Biol.

[B31] Boffelli D, McAuliffe J, Ovcharenko D, Lewis KD, Ovcharenko I, Pachter L, Rubin EM (2003). Phylogenetic shadowing of primate sequences to find functional regions of the human genome. Science.

[B32] Kellis M, Patterson N, Endrizzi M, Birren B, Lander ES (2003). Sequencing and comparison of yeast species to identify genes and regulatory elements. Nature.

[B33] Kronforst MR, Kapan DD, Gilbert E (2006). Parallel genetic architecture of parallel adaptive radiations in mimetic *Heliconius *butterflies. Genetics.

[B34] Vos P, Hogers R, Bleeker M, Reijans M, Lee Tvd, Hornes M, Frijters A, Pot J, Peleman J, Kuiper M (1995). AFLP: a new technique for DNA fingerprinting. Nucleic Acids Res.

[B35] Papa R, Troggio M, Ajmone MP, Nonnis MF (2005). An improved protocol for the production of AFLP markers in a complex genomes by means of capillary electrophoresis. J Anim Breed Genet.

[B36] Michelmore RW, Paran I, Kesseli RV (1991). Identification of markers linked to disease-resistance genes by bulked segregant analysis: A rapid method to detect markers in specific genomic regions by using segregating populations. Proc Natl Acad Sci USA.

[B37] Turner JRG, Smiley J (1975). Absence of crossing-over in female butterflies (*Heliconius*). Heredity.

[B38] Suomalainen E, Cook LM, Turner JRG (1973). Achiasmatic oogenesis in the heliconiine butterflies. Hereditas.

[B39] Brugmans B, Hulst RGM Van der, Visser RGF, Lindhout P, Van Eck HJ (2003). A new and versatile method for the successful conversion of AFLP markers into simple single locus markers. Nucleic Acids Res.

[B40] Wu C, Xu Z, Zhang H-B, Meyers RA (2004). DNA Libraries. Encyclopedia of Molecular Cell Biology and Molecular Medicine.

[B41] Nickerson DA, Tobe VO, Taylor SL (1997). PolyPhred: automating the detection and genotyping of single nucleotide substitutions using fluorescence-based resequencing. Nucleic Acids Res.

[B42] Bonfield JK, Smith K, Staden R (1995). A new DNA sequence assembly program. Nucleic Acids Res.

[B43] KAIKOGAAS: an automated annotation system designed for analysis of the silkworm genome. http://kaikogaas.dna.affrc.go.jp/.

[B44] Shimomura M, Shimizu Y, Sasanuma S-i, Antonio BA, Nagamura Y, Mita K, Sasaki T KAIKOGAAS: An automated annotation System for silkworm genome. Genome Inform.

[B45] Papanicolaou A, Gebauer-Jung S, Blaxter ML, McMillan WO, Jiggins CD (2008). ButterflyBase: a platform for lepidopteran genomics. Nucl Acids Res.

[B46] Altschul SF, Gish W, Miller W, Myers WE, Lipman DJ (1990). Basic local alignment search tool. J mol biol.

[B47] Brudno M, Do CB, Cooper GM, Kim MF, Davydov E, Green ED, Sidow A, Batzoglou S, NISC Comparative Sequencing Program (2003). LAGAN and Multi-LAGAN: efficient tools for large-scale multiple alignment of genomic DNA. Genome Res.

[B48] Volfovsky N, Haas BJ, Salzberg SL (2001). A clustering method for repeat analysis in DNA sequences. Genome Biol.

[B49] Ihaka R, Gentleman R (1996). A language for data analysis and graphics. J Comput Graph Statist.

